# Metabolic Implications when Employing Heavy Pre- and Post-Exercise Rapid-Acting Insulin Reductions to Prevent Hypoglycaemia in Type 1 Diabetes Patients: A Randomised Clinical Trial

**DOI:** 10.1371/journal.pone.0097143

**Published:** 2014-05-23

**Authors:** Matthew D. Campbell, Mark Walker, Michael I. Trenell, Steven Luzio, Gareth Dunseath, Daniel Tuner, Richard M. Bracken, Stephen C. Bain, Mark Russell, Emma J. Stevenson, Daniel J. West

**Affiliations:** 1 Department of Sport, Exercise and Rehabilitation, Faculty of Health and Life Sciences, Northumbria University, Newcastle-upon-Tyne, United Kingdom; 2 Institute of Cellular Medicine, Newcastle University, Newcastle-upon-Tyne, United Kingdom; 3 Institute for Ageing and Health, Faculty of Medicine, Newcastle University, Newcastle-upon-Tyne, United Kingdom; 4 Diabetes Research Group, College of Medicine, Swansea University Swansea, United Kingdom; 5 Applied Sports, Technology, Exercise and Medicine Research Centre, College of Engineering, Swansea University, Swansea, United Kingdom; NIDDK/NIH, United States of America

## Abstract

**Aim:**

To examine the metabolic, gluco-regulatory-hormonal and inflammatory cytokine responses to large reductions in rapid-acting insulin dose administered prandially before and after intensive running exercise in male type 1 diabetes patients.

**Methods:**

This was a single centre, randomised, controlled open label study. Following preliminary testing, 8 male patients (24±2 years, HbA1c 7.7±0.4%/61±4 mmol.l^−1^) treated with insulin's glargine and aspart, or lispro attended the laboratory on two mornings at ∼08:00 h and consumed a standardised breakfast carbohydrate bolus (1 g carbohydrate.kg^−1^BM; 380±10 kcal) and self-administered a 75% reduced rapid-acting insulin dose 60 minutes before 45 minutes of intensive treadmill running at 73.1±0.9% VO_2peak_. At 60 minutes post-exercise, patients ingested a meal (1 g carbohydrate.kg^−1^BM; 660±21 kcal) and administered either a **Full** or **50%** reduced rapid-acting insulin dose. Blood glucose and lactate, serum insulin, cortisol, non-esterified-fatty-acids, β-Hydroxybutyrate, and plasma glucagon, adrenaline, noradrenaline, IL-6, and TNF-α concentrations were measured for 180 minutes post-meal.

**Results:**

All participants were analysed. All glycaemic, metabolic, hormonal, and cytokine responses were similar between conditions up to 60 minutes following exercise. Following the post-exercise meal, serum insulin concentrations were lower under **50%** (*p*<0.05) resulting in 75% of patients experiencing hyperglycaemia (blood glucose ≥8.0 mmol.l^−1^; **50%** n = 6, **Full** n = 3). β-Hydroxybutyrate concentrations decreased similarly, such that at 180 minutes post-meal concentrations were lower than rest under **Full** and **50%**. IL-6 and TNF-α concentrations remained similar to fasting levels under **50%** but declined under **Full**. Under **50%** IL-6 concentrations were inversely related with serum insulin concentrations (*r* = −0.484, *p* = 0.017).

**Conclusions:**

Heavily reducing rapid-acting insulin dose with a carbohydrate bolus before, and a meal after intensive running exercise may cause hyperglycaemia, but does not augment ketonaemia, raise inflammatory cytokines TNF-α and IL-6 above fasting levels, or cause other adverse metabolic or hormonal disturbances.

**Trial Registration:**

ClinicalTrials.gov NCT01531855

## Introduction

Cardiorespiratory or endurance-based exercise is promoted as playing an integral role in maintaining a healthy lifestyle for patients with type 1 diabetes because of its utility to enhance cardiovascular fitness, improve anthropometrics and reduce total daily insulin requirements (see Chu et al [1]). Although this form of exercise carries blood glucose lowering effects which may initially offer some therapeutic benefit, reductions in blood glucose following exercise are often excessive, exposing patients to hypoglycaemia [2]. To reduce the incidence of hypoglycaemia during and immediately after exercise, patients are recommended to reduce their pre-exercise rapid-acting insulin dose [2], [3]. However, recent evidence also suggests that it is important for patients to reduce the dose of rapid-acting insulin administered with the meal after exercise as well, so that the risk of post-exercise hypoglycaemia is abated [4].

In combining pre and post-exercise rapid-acting insulin dose reductions some patients may be exposed to periods of hyperglycaemia [4]. With this is mind, it is reasonable to speculate that low levels of circulating insulin combined with elevated concentrations of post-exercise counter-regulatory hormones may in fact precipitate a metabolic milieu promoting increased lipolysis [5] and ketogenesis [6]. In addition, inflammatory cytokine responses are related to hyperglycaemia [7]–[10], lipid oxidation [11], and/or hyperketonaemia [12]–[14]. Although regular exercise has been demonstrated to reduce systemic inflammation, thus strengthening its therapeutic utility for patients with type 1 diabetes [15], somewhat paradoxically, these long-term adaptations occur despite opposing acute effects in which there is a pronounced increase in inflammatory markers early after exercise [16]–[21].

Potentially, performing exercise under conditions of concurrent or prior hyperglycaemia and/or hypoinsulinaemia might result in an inappropriately elevated level of inflammation after exercise. Additionally, metabolic disturbances could negate the over-all health benefits of exercise and promote the onset and progression of diabetic complications. Indeed, this may offer some explanation towards conflicting opinion regarding the efficacy of aerobic or endurance-based exercise for improvements in glycaemic control, with some literature demonstrating no improvement or even worsening HbA1c (for review see Tonoli et al [22]).

It is a well-established recommendation that patients reduce their pre-exercise rapid-acting insulin dose to prevent exercise-induced hypoglycaemia, and the metabolic implications of this are well known [23]. However, the deeper metabolic consequences of employing the recently advocated strategy of heavily reducing both pre and post-exercise rapid-acting insulin dose [4] are unknown. Therefore, the aim of this study was to determine whether reducing pre- and also post-exercise rapid-acting insulin dose, as a strategy for preventing post-exercise hypoglycaemia, causes metabolic or hormonal disturbances, influences ketonaemia, or alters inflammatory cytokine concentrations in type 1 diabetes patients, following intensive running exercise.

## Methods

### Ethics statement

This study was approved by local National Health Service Research Ethics Committee (13/NE/0016); registry details (NCT01531855, ClinicalTrials.gov). All patients who participated provided full written informed consent. All clinical investigations were conducted according to the principles expressed in the Declaration of Helsinki. The study was conducted between 1^st^ March 2013 and 1^st^ September 2013.

### Protocol

The protocol for this trial and supporting CONSORT checklist are available as supporting information; see Checklist S1 and Protocol S1. Eight male type 1 diabetes patients (mean ± SEM; age 24±2 years, BMI 22.9±0.7, duration of diabetes 13±2 years, HbA_1c_ 7.7±0.4%/61±4 mmol.l^−1^, 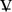
O_2peak_ 54±1 ml.kg.min^−1^) volunteered to participate. Female participants were not included due to the potential influence of the menstrual cycle on blood glucose homeostasis [24]. Interested patients who met the eligibility criteria for involvement in the study (aged between 18–35 years with a duration of diabetes greater than 2 years on enrolment, treated with a stable basal-bolus insulin regimen composed of insulin glargine and fast-acting insulin aspart or lispro for a minimum of 6 months, free of diabetes related complications, and receiving no additional medication other than insulin) were screened in-line with the procedures of American College of Sports Medicine's (ACSM) Guidelines for Exercise Testing and Prescription [25]. Screening measures included a comprehensive medical questionnaire and the completion of a cardiopulmonary exercise stress test to assess cardiac function in response to exercise. All patients were regularly and consistently active (self-reporting to participate in aerobic based exercise for at least 30 minutes at a time, 3 times per week), and displayed normal cardio-pulmonary responses to exercise. All patients were familiar with carbohydrate counting. Patients underwent randomisation using a computer programme to determine the sequence of two open label crossover arms.

Upon completion of the screening procedures, patients completed a preliminary incremental treadmill test to quantify peak cardio-respiratory parameters as described previously by our group [2], [4]. Following this, patients attended the Newcastle NIHR Clinical Research Facility exercise laboratory on two occasions, once per week at ∼08:00 h. Patients replicated their diet (assessed using weighed dietary recording sheets) and maintained a similar insulin regimen (with basal insulin dose standardised i.e. dose, injection site, time of injection) during the 24 hours preceding each trial. Additionally, patients were required to avoid exercise for 24 hours prior to each visit; activity patterns were measured using a pedometer (Omron Healthcare Europe B.V., Netherlands). Patients maintained their usual basal regimen (dose, injection site, time of injection) between trials.

Patients arrived at the laboratory after an overnight fast; if patients experienced a hypoglycaemic episode on the preceding night their trial was rescheduled. Following their arrival, patients assumed a resting position while a 20 gauge cannula (Vasofix, B.Braun Melsungen AG, Germany) was inserted into the antecubital vein of their non-dominant arm. An 11 ml resting venous blood sample was taken, of which 20 µl was analysed immediately for glucose (BG) and lactate (Biosen C-Line, EKF Diagnostic GmbH, Germany), and 10 µl analysed for haemoglobin and haematocrit (Hemo Control, EKF-diagnostic GmbH, Germany) which was used to correct for changes in plasma volume [26]. The remaining sample was dispensed evenly into Lithium-heparin tubes (Vacuette, Greiner Bio-One GmBH, Austria) and serum separation tubes (Vacuette, Greiner Bio-One GmBH, Austria) which were centrifuged for 15 minutes at 3000 rev.min^−1^, and stored at −80°C for later analysis of rapid-acting insulin (Invitron insulin assay, Invitron, UK), glucagon (Glucagon EIA, Sigma-Aldrich, USA), adrenaline and noradrenaline (BI-CAT adrenaline and noradrenaline ELISA, Eagle Biosciences, UK), cortisol (Parameter cortisol ELISA, R & D Systems, Roche Diagnostics, UK), non-esterified-fatty-acids (NEFA; Non-esterified fatty acids colorimetric assay, Randox Laboratories, UK), β-Hydroxybutyrate (D-3-Hydroxybutyrate kinetic enzymatic assay, Randox Laboratories, UK), interleukin-6 (IL-6; Human IL-6 Quantikine ELISA, R & D Systems, Roche Diagnostics, UK) and tumour necrosis factor alpha (TNF-α; Human TNF-alpha Quantikine ELISA, R & D Systems, Roche Diagnostics, UK). The coefficient of variation was <10% for all assays.

Following the resting sample, patients were provided with a standardised cereal-based breakfast-carbohydrate bolus (frosted flakes, semi-skimmed milk, peaches) equating to 1 g carbohydrate.kg^−1^BM (380±10 kcal; 1.59±0.04 MJ). Immediately before consumption, patients administered a 25% (1.8±0.1 IU) dose (i.e. a 75% reduction) of rapid-acting insulin into the abdomen, as per current recommendations [2], [3]. This pre-exercise carbohydrate and insulin strategy was based on previously published methodologies [3], [27]. All patients were administering 1.0±0.1 IU of rapid-acting insulin per 10 g of carbohydrate. The injection site was standardised across trials through administration at half the distance between the anterior-superior iliac crest and the naval. Insulin aspart and lispro were administered using Novopen3 (NovoNordisk, UK) and Humapen luxura (Eli Lilly, UK), respectively.

A further blood sample was collected at 60 minutes following the ingestion of the breakfast meal. At 60 minutes, patients performed a 45 minute bout of treadmill running (Woodway, Germany) at a velocity calculated to elicit 70%VO_2peak_. During exercise, breath-by-breath respiratory parameters (MetaLyzer 3B; Cortex, Germany) and heart rate (S810; Polar, Finland) were continuously recorded. Immediately upon cessation of exercise, a blood sample was obtained with further blood samples collected at 15, 30, and 60 minutes post-exercise. At 60 minutes post-exercise, patients were provided with a pasta-based lunch (pasta, tomato based sauce, cheddar cheese) equating to 1 g carbohydrate.kg^−1^BM (660±21 kcal; 2.76±0.09 MJ) to which they administered either a **Full** (7.5±0.3 IU) or **50%** (3.7±0.1 IU) rapid-acting insulin dose immediately before consumption, into the contralateral abdominal site to that which received the pre-exercise rapid-acting insulin dose. Having consumed the meal, patients remained rested for 180 minutes with periodic blood samples every 30 minutes. Hypoglycaemia was defined as a blood glucose concentration of ≤3.9 mmol.1^−1^ and hyperglycaemia as ≥8.0 mmol.1^−1^. If patients experienced hypoglycaemia during the laboratory period a 20 g carbohydrate bolus was administered (Lucozade, GlaxoSmithKline, UK). Hyperketonaemia was defined as a β-hydroxybutyrate concentration >1.0 mmol.l^−1^ [6].

### Data analysis

Sample size was calculated using data from Campbell et al [4] and the methods of Hopkins [28] whereby the number of participants required is based around the precision defined by 95% confidence limits (deriving a power 0.8). The sample size is calculated as *n*  = 8*s^2^*/*d^2^* where *n* is the samples size, *s* is the typical error, and *d* is the smallest worthwhile effect. A sample size of 8 participants was required to achieve 80% power. Statistical analysis was performed using PASW *Statistics* software (IBM PASW version 18; IBM., NY, USA) with significance set at *p*≤0.05. Interactions of time and condition were examined using repeated measures ANOVA. Where significant *p*-values were identified for interaction effects (time*condition), insulin dose was deemed to have influenced the response, and simple main effects analyses were performed. Significant main effects of time were further investigated using BONFERRONI adjusted pairwise comparisons. One-way repeated ANOVA were performed where relevant and relationships were explored using Pearson's product moment correlation coefficient. Data are presented as mean ± SEM.

## Results

**Figure 1:**
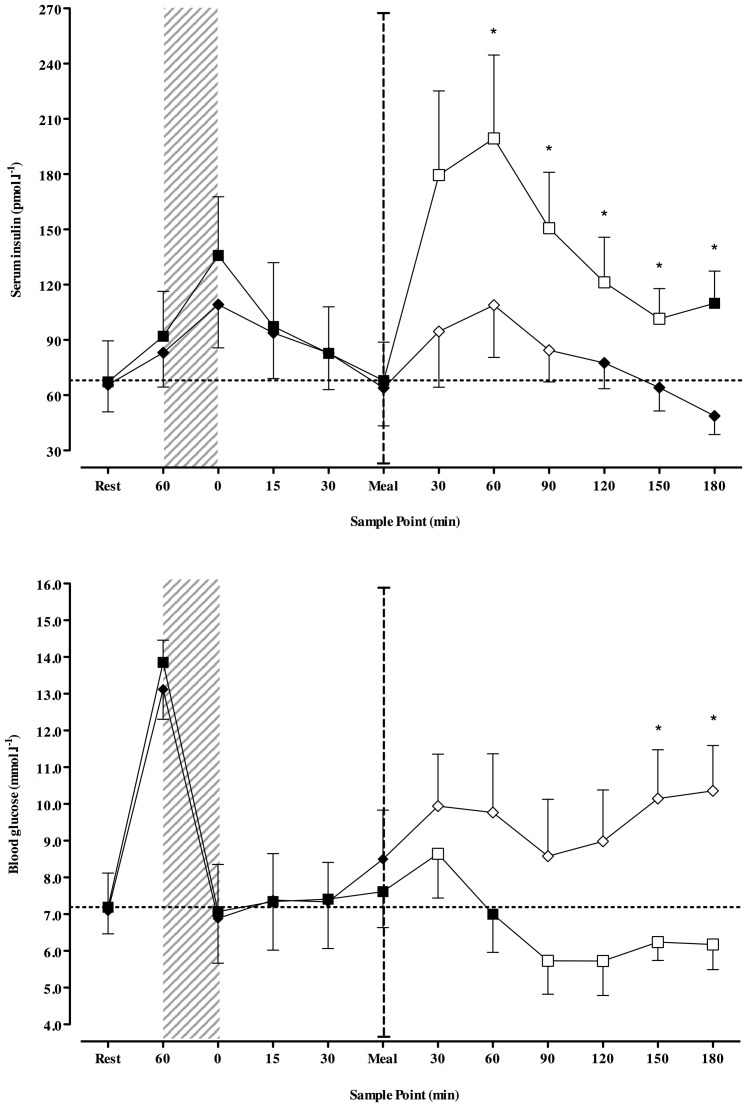
A–B. Time-course changes in serum insulin (A) and blood glucose (B) from rest. Full  =  black squares, 50%  =  black diamond.* indicates significantly different from Full (*p*<0.05). Transparent sample point within a condition indicates a significant difference from pre-exercise meal concentrations (*p*<0.05). Vertical dashed line break indicates post-exercise intervention. NOTE: test meal and insulin were administered immediately following rest and meal sample points.

All patients were analysed for this study. A significant interaction of condition and time were evident when examining serum insulin (*p*<0.001) and blood glucose (condition*time, *p* = 0.014). Serum insulin (Figure 1A) and blood glucose (Figure 1B) concentrations were similar between conditions at baseline and immediately before exercise. The performance of exercise was intensive, with patients exercising at a similar % of 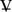
O_2peak_ (**Full** 74.5±0.01; **50%** 71.6±0.02% 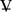
O_2peak_, *p* = 0.329), and HR_peak_ (**Full** 80±2; **50%** 79±1%HR_peak_, *p* = 0.785). The average respiratory exchange ratio during exercise was similar between conditions (**Full** 0.98±0.01; **50%** 0.96±0.02, *p* = 0.256), resulting in similar reductions in blood glucose (**Full** −7.8±0.8; **50%** −7.5±1.11 mmol.l^−1^, *p* = 0.453, Figure 1B) and similar peaks in blood lactate. Serum insulin (Figure 1A) and blood glucose (Figure 1B) concentrations up to 60 minutes post-exercise were similar between conditions, such that blood glucose was comparable immediately prior to the administration of the post-exercise meal (Figure 1B).

Following the post-exercise meal, serum insulin peaked at 60 minutes under both conditions, with mean peak concentrations greater under **Full** (199±47; **50%** 109±28 pmol.l^−1^, *p* = 0.021; Figure 1A). From 60 minutes post-meal, serum insulin under **Full** remained elevated from pre-meal measures, and significantly higher than **50%** (*p*<0.05; Figure 1A). Blood glucose declined under **Full** (Figure 1B), but was preserved under **50%** with concentrations greater than those at pre-meal throughout the three hour observation period (Figure 1B). During this time, 63% of patients (n = 5) experienced hypoglycaemia (BG≤3.9 mmol.l^−1^) under **Full**, whereas all patients under **50%** were protected. Inversely, fewer patients under **Full** experienced hyperglycaemia (BG≥8 mmol.l^−1^; **Full** n = 3; **50%** n = 6).

There were no conditional differences in counter-regulatory hormones (Table 1) or metabolites (Table 2) up to 60 minutes post-exercise (*p*>0.05). A significant interaction of condition and time (*p* = 0.006) were found when examining plasma glucagon concentrations during the trials (Table 2). Plasma glucagon concentrations following the post-exercise meal were significantly elevated from pre-meal values under both conditions, but were highest under **Full** (Table 1). There were no differences in β-Hydroxybutyrate, adrenaline, noradrenaline, cortisol, blood lactate, or NEFA concentrations between conditions following the post-exercise meal (Table 1 and 2). During this time, there was a transient decline in β-Hydroxybutyrate with concentrations similar to rest under **50%** and lower than rest under **Full** (Table 2).

**Table 1:** Counter-regulatory responses to reductions in pre- and post-exercise rapid-acting insulin dose during the experimental trials.

															ANOVA *p*	
		Rest	60	Exercise	0	15	30	Pre-Meal	30	60	90	120	150	180	Time	Time*Condition
**Plasma Glucagon (pg.ml^−1^)**	**Full**	760±70	697±81	-	1152±99†	832±83†	698±61	710±69	1015±79† ‡	1597±116† ‡	1590±102† ‡	1402±94† ‡	1332±137† ‡	1099±124† ‡	<0.001	= 0.006
	**50%**	789±85	628±85† *	-	1067±128†	854±127	815±89	609±98†	975±82† ‡	1350±96† * ‡	1251±97† * ‡	1177±90† * ‡	1085±86† * ‡	889±82* ‡		
**Plasma Adrenaline (nmol**.**l^−1^)**	**Full**	0.30±0.05	0.14±0.04†	-	0.63±0.11†	0.49±0.12	0.37±0.08	0.26±0.06	0.24±0.06	0.23±0.10	0.14±0.05†	0.19±0.06	0.21±0.07	0.16±0.05† ‡	<0.001	= 0.808
	**50%**	0.30±0.07	0.21±0.05	-	0.68±0.08†	0.34±0.05	0.23±0.05	0.16±0.04	0.16±0.05	0.17±0.04	0.16±0.05	0.17±0.06	0.16±0.04	0.15±0.04		
**Plasma Noradrenaline (nmol**.**l^−1^)**	**Full**	2.11±0.33	1.72±0.27	-	12.98±1.56†	4.76±0.69†	3.15±0.36†	2.59±0.36	2.65±0.33	2.72±0.46	2.36±0.37	2.39±0.48	2.30±0.42	2.21±0.43	<0.001	= 0.407
	**50%**	2.25±0.34	2.17±0.32	-	13.63±1.84†	4.16±0.35†	3.34±0.25†	2.71±0.34	2.67±0.52	2.87±0.61	2.62±0.47	2.90±0.73	2.92±0.68	2.77±0.61		
**Serum Cortisol (nmol**.**l^−1^)**	**Full**	0.23±0.04	0.22±0.03†	-	0.21±0.03†	0.25±0.03	0.22±0.03	0.18±0.02†	0.18±0.03†	0.16±0.03† ‡	0.12±0.02† ‡	0.12±0.03†	0.10±0.01†	0.10±0.01†	= 0.019	= 0.394
	**50%**	0.22±0.04	0.18±0.05†	-	0.20±0.03	0.24 ±0.04	0.22±0.03	0.18±0.02	0.15±0.02	0.12±0.02†	0.13±0.03†	0.11±0.02	0.09±0.01	0.07±0.01		

NOTE: Data presented as mean ± SEM. Test meal and insulin were administered immediately following rest and pre-meal sample points.

* indicates significantly different from Full (*p*≤0.05).

† indicates significantly different from rest.

‡ indicates significantly different from pre-meal.

NOTE: Exercise commenced 60 minutes after rest.

**Table 2:** Metabolic responses to reductions in pre- and post-exercise rapid-acting insulin dose during the experimental trials.

															ANOVA *p*	
		Rest	60	Exercise	0	15	30	Pre-Meal	30	60	90	120	150	180	Time	Time*Condition
**Blood Lactate (mmol.l^−1^)**	**Full**	0.38±0.14	0.72±0.15	-	3.63±0.62†	1.30±0.16†	0.93±0.11†	0.69±0.20†	0.49±0.17	0.56±0.13	0.40±0.14	0.38±0.13	0.35±0.13‡	0.37±0.13	= 0.001	= 0.312
	**50%**	0.47±0.15	0.75±0.12	-	3.60±0.43†	1.38±0.23†	0.77±0.18	0.60±0.16	0.43±0.18	0.53±0.13	0.46±0.15	0.43±0.14	0.42±0.13	0.35±0.11		
**Serum NEFA (mmol.l^−1^)**	**Full**	0.41±0.07	0.22±0.04†	-	0.24±0.04†	0.35±0.18	0.32±0.05	0.34±0.07	0.26±0.04†	0.20±0.04† ‡	0.18±0.04† ‡	0.26±0.04†	0.25±0.03†	0.25±0.02†	= 0.019	= 0.394
	**50%**	0.36±0.06	0.18±0.04†	-	0.17±0.02	0.26±0.05	0.15±0.04	0.24±0.06†	0.24±0.05†	0.16±0.04†	0.19±0.03†	0.33±0.03	0.39±0.05	0.44±0.05		
**Serum β-OH (mmol.l^−1^)**	**Full**	0.11±0.03	0.03±0.01†	-	0.08±0.01	0.08±0.02	0.09±0.01	0.06±0.01	0.06±0.01	0.04±0.01†	0.05±0.01	0.08±0.03	0.04±0.01†	0.04±0.01†	= 0.046	= 0.303
	**50%**	0.08±0.02	0.05±0.01†	-	0.06±0.01	0.07±0.01	0.05±0.01	0.04±0.01	0.11±0.05	0.06±0.03	0.03±0.01†	0.04±0.03†	0.04±0.03†	0.06±0.01		

NOTE: Data presented as mean ± SEM. β-OH  =  β-Hydroxybutyrate. Test meal and insulin were administered immediately following rest and pre-meal sample points.

* indicates significantly different from Full (*p*≤0.05).

† indicates significantly different from rest.

‡ indicates significantly different from pre-meal.

NOTE: Exercise commenced 60 minutes after rest.

**Figure 2:**
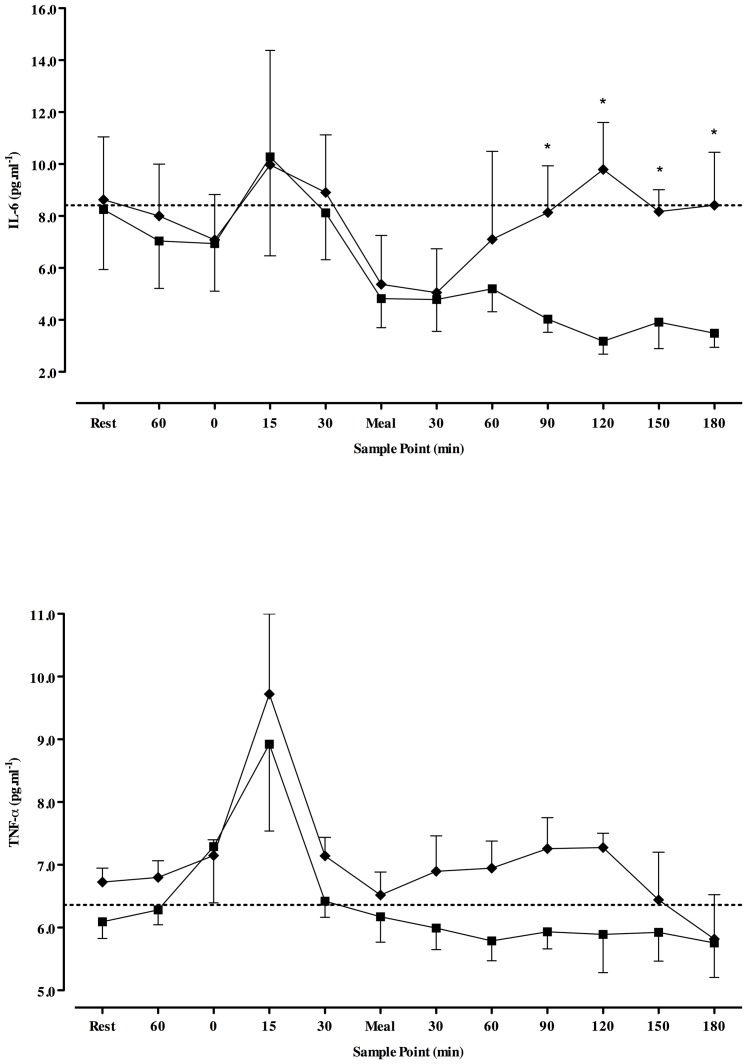
A–B. Time-course changes in IL-6 (A) and TNF-α (B) from rest. Full  =  black squares, 50%  =  black diamond.* indicates significantly different from Full (*p*<0.05). Transparent sample point within a condition indicates a significant difference from pre-exercise meal concentrations (*p*<0.05). Vertical dashed line break indicates post-exercise intervention. NOTE: test meal and insulin were administered immediately following rest and meal sample points.

The IL-6 and TNF-α responses are presented in Figure 2 A–B. Plasma TNF-α concentrations were significantly raised at 15 minutes post-exercise. Following the post-exercise meal, IL-6 concentrations were significantly greater under **50%**. However, TNF-α was similar between conditions. Despite this, both cytokines under **50%** remained similar to pre-meal and resting measures (*p*>0.05; Figure 2A–B). Under **50%** mean IL-6 concentrations over the post-exercise period were positively related to mean TNF-α concentrations (*r* = 0.676, *p*<0.001) and serum insulin concentrations were inversely related to IL-6 (*r* = −0.484, *p* = 0.017), but not TNF-α (*r* = −0.169, *p* = 0.430). No significant relationships existed between mean blood glucose and IL-6 (*r* = 0.299, *p* = 0.155) or TNF-α (*r* = 0.005, *p* = 0.980) over the post-meal period under **50%**. No relationships were found between any measures under **Full**.

## Discussion

To our knowledge we are the first to demonstrate, that manipulating pre- and also post-exercise rapid-acting insulin dose, as a strategy for preventing post-exercise hypoglycaemia does not cause adverse metabolic or counter-regulatory-hormonal disturbances yet patients remain protected from hypoglycaemia. Specifically, our data indicate that large reductions in rapid-acting insulin dose administered before and also after intensive running exercise may cause post-exercise hyperglycaemia, but this strategy does not augment ketonaemia in patients with type 1 diabetes. In addition, we demonstrate that inflammatory cytokines may increase with this strategy, but not above fasting concentrations, and by 180 minutes post-meal the pro-inflammatory cytokine TNF-α is lower than rest.

Patients completed an intensive bout of prolonged running exercise, running at an average speed of 9.3±0.3 km.hr^−1^ and covering a distance of 7.0±0.2 km. The respiratory exchange ratio (∼0.96) exhibited by patients is typical of exercise of an intense nature [29], and reflects the predominant use of carbohydrate during exercise. Indeed, completing the exercise protocol caused a significant metabolic stress to patients, inducing large increases in blood lactate (∼392%) and catecholamines (adrenaline **∼**287%, noradrenaline **∼**591%), and large decreases in blood glucose (Δ∼7.6 mmol.l^−1^). Despite subsequent reductions in glycaemia, and the administration of a second dose of rapid-acting insulin 60 minutes post-exercise, all patients under **50%** were protected from hypoglycaemia throughout their laboratory stay. As such, we demonstrate that administering a small dose of rapid-acting insulin with a carbohydrate bolus before exercise is effective in limiting pre-exercise hyperglycaemia and preventing hypoglycaemia during and immediately after exercise. Importantly, circulating insulin concentrations following exercise returned to baseline, meaning the effects of administering a pre-exercise rapid-acting insulin dose of this size is unlikely to carry additive effects for subsequent post-exercise rapid-acting insulin administration. Therefore, reducing pre- and also post-exercise rapid-acting insulin dose is an effective strategy for preventing exercise-induced hypoglycaemia. However, preserving glycaemia consequently exposed patients under **50%** to periods of hyperglycaemia following the post-exercise meal. This finding emphasises that patients differ in their sensitivity to insulin following exercise, and as such, we advise that patients may require a smaller reduction in post-exercise rapid-acting insulin dose.

Although the majority of patients under **50%** were exposed to hyperglycaemia, β-Hydroxybutyrate concentrations following the post-exercise meal remained similar between both conditions, with values remaining less than those considered hyperketonaemic (<1.0 mmol.l^−1^) [6] and similar to rest. Although we applied a large reduction in rapid-acting insulin dose, serum insulin concentrations were elevated above resting and pre-meal measures under **50%** (Table 1). Despite unexplained differences in glucagon concentrations, the administration of even small amounts of rapid-acting insulin, under conditions of unchanged basal insulin dose, is likely to have raised circulating insulin concentrations to a level where β-Hydroxybutyrate production was suppressed [30], and potentially augment peripheral ketone body disposal [31]. Additionally, NEFA concentrations did not increase, likely attributable to unchanged concentrations of catecholamines, the main lipolytic stimulus [32], and cortisol [33]. Furthermore, the consumption of a large carbohydrate based meal would have helped supplement muscle and liver glycogen, reducing the energy deficit created by exercise, and limiting the appearance of catecholamines and cortisol.

We questioned whether post-exercise hyperglycaemia would exacerbate the appearance of inflammatory cytokines in our patients, indeed this may be further exacerbated if patients experienced hyperketonaemia. Hyperglycaemia associated inflammation is a major contributor to the pathogenesis of diabetes related complications [9], [33], which are primarily avoided through the normalisation of glycaemic profiles [34]. Patients with type 1 diabetes exhibit chronically elevated levels of inflammatory markers at baseline [7]–[10], [35]. Baseline measures were elevated above some of those previously reported [7], [35]–[38], which may have resulted from patients arriving in a fasted state and with low circulating concentrations of insulin. However, comparison between studies is hampered by mixed methodologies and differing patient characteristics, indeed most studies implement glucose and/or insulin clamp procedures, and recruit children or adolescents who are usually recently diagnosed [7], [35]–[38]. Our study population consisted of a relatively young (∼24 years) group of individuals all in good glycaemic control (∼7.7%/61 mmol.l^−1^), exposure to inflammatory stimuli is likely to be much greater in the general diabetes population who are older, have a longer duration of diabetes, and in those with excess adiposity [10].

We witnessed only modest increases in IL-6 (∼22%) and TNF-α (∼45%) following exercise, which is likely due to the administration of the pre-exercise meal and concomitant insulin in our patients. The large carbohydrate bolus (1.0 g carbohydrate.kg^−1^ BM) would have helped supplement glycogen reserves [39], which may have attenuated the exercise-induced increases in IL-6 [12], [40] and even completely inhibited IL-6 release from contracting skeletal muscle [40]. In addition, insulin carries anti-inflammatory properties [41], of which its administration, even in small doses, may have partially combatted the pro-inflammatory effects of TNF-α. Indeed, IL-6 concentrations were inversely related to circulation insulin concentrations. IL-6 has anti- as well as pro-inflammatory properties, with some studies demonstrating IL-6 to exert inhibitory effects on TNF-α. We found there to be a positive correlation between IL-6 and TNF-α. Although a relationship does not necessarily indicate cause-effect, speculatively, increases in IL-6 may indeed have been in direct response to reducing TNF-α. Irrelevant of the mechanisms at play, our data indicate that TNF-α or IL-6 are not significantly elevated above fasting concentrations, and that by 180 minutes post-meal, concentrations are lower than those following an overnight fast.

We chose IL-6 and TNF-α as our main inflammatory markers, in part, because both display the greatest quantitative change [40] and therefore have a high likelihood to yield distinct differences between our study conditions [16], [20], [35], [37]. There is however, a known and marked inherent variability of many inflammatory markers [7], [37], which reflects the remarkable metabolic complexity of that patient with type 1 diabetes in which permutations in inflammation status are variable across patients and within the same individuals overtime [7]. Indeed, some of this variability is attributed to antecedent hyperglycaemia [7], [16]–[21], [36], [38], however patients in this study were kept under free-living conditions before experimentation and without correction using euglycaemic clamp procedures, and therefore likely to closely replicate the responses in patients in day-to-day life. It would be inappropriate to conclude that inflammation *per se* was unchanged in our patients, as we were only able to analyse two inflammatory cytokines within our dataset. Although TNF-α and IL-6 are readily accepted as primary markers of inflammation, future research investigating more detailed post-prandial inflammatory responses in this population is warranted.

An interesting finding was that plasma glucagon concentrations following the post-exercise meal were elevated under both conditions, and significantly greater under **Full**. Although the majority of patients under this condition experienced hypoglycaemia (BG≤3.9 mmol.l^−1^; **50%** n = 0, **Full** n = 5), patients were treated with a corrective bolus of carbohydrate, such that blood glucose levels (group mean BG∼6.6 mmol.l^−1^) remained above the glycaemic threshold for plasma glucagon release (BG<3.0 mmol.l^−1^; [42]; even if an appropriate glycaemic threshold was achieved to stimulate glucagon release, it would remain a surprise to find any increase in glucagon concentrations in our patients, as its secretion under exercising and hypoglycaemic conditions is blunted in type 1 diabetes [42]. In addition, increased glucagon response to a mixed-meal stimulus has been noted previously in type 1 diabetes [43], suggestive that the α-cell secretory reserve may be unaffected by the progression of the autoimmune process [43]. Of note, the meal administered in the study by Brown et al [43], was similar in nutritional content to the post-exercise meal given to patients in our study (Carbohydrate 56 vs. 53%; Protein 21 vs. 25%, Fat 21 vs. 22%), although our meal was larger (400 kcal vs. ∼660 kcal). Considering all of our patients had longstanding diabetes (length of disease ∼13 years), were intensively controlled (∼7.7%/61 mmol.l^−1^), and therefore likely to have been frequently exposed to hypoglycaemia, investigation of differing types of post-exercise meal may be an important avenue to investigate as altering the composition or glycaemic index of the post-exercise meal may indeed offer more favourable glycaemic profiles by reducing the incidence of hyperglycaemia.

To conclude, heavily reducing pre- and post-exercise rapid-acting insulin dose does not induce hyperketonaemia, excessively raise inflammatory cytokines TNF-α or IL-6, or cause other metabolic or hormonal disturbances in type 1 diabetes patients treated with insulin glargine and fast-acting insulin aspart or lispro. In addition, we further highlight the importance of this strategy in preventing early-onset post-exercise hypoglycaemia. However, patients may be exposed to hyperglycaemia and are therefore encouraged to regularly monitor blood glucose concentrations after exercise, and refine these strategies such that euglycaemia can be achieved following individual exercise routines. We advise that future research investigates in more detail post-prandial inflammatory responses in this population.

## Supporting Information

Checklist S1 **CONSORT checklist.**
(DOCX)

Protocol S1 **Study protocol.**
(DOC)

Diagram S1 (DOC)
