# Supplementary material for: Metabolic Implications when Employing Heavy Pre- and Post-Exercise Rapid-Acting Insulin Reductions to Prevent Hypoglycaemia in Type 1 Diabetes Patients: A Randomised Clinical Trial
Source: PLoS One. 2014 May 23;9(5):e97143. doi: 10.1371/journal.pone.0097143 (PMC4032262; doi:10.1371/journal.pone.0097143)
Supplement: Protocol S1 — Study protocol. (DOC) [file pone.0097143.s002.doc]

**Design:**

8 male T1DM individuals aged between 18 and 50 years old will be invited to attend four laboratory sessions, each separated by 7 days. After two preliminary visits, they will complete two main trials in a randomised and counter balanced fashion. Each main trial will involve completing a fixed bout of running having administered a reduced rapid acting insulin dose with a standardised carbohydrate based meal, 60 minutes before exercise. Sixty minutes after exercise participants will administer a normal, or 50% reduced rapid acting insulin dose with a standardised carbohydrate meal. Metabolic, glycaemic and inflammatory responses will be measured before and for 3 hours after exercise

**Inclusion:**

For inclusion in the study, volunteers will be male and aged 18-50 years old, free from any diabetes complications apart from background diabetic retinopathy, not taking any prescribed medication other than insulin, and be treated with a stable insulin regimen composed of a combination of slow/long acting insulin (glargine) and fast acting insulin analogues (lispro or aspart) for 6 months before the start of the study, and have a HbA1c of 6.5 - 8.5%. Volunteers meeting these criteria will then attend the laboratory for an electrocardiogram exercise stress test. If normal cardiac function is confirmed, volunteers will then proceed to participate in the study.

**Preliminary Testing:**

The preliminary testing consists of an initial visit to the laboratory to establish anthropometric characteristics of the participants (body mass, stature, body fat %, waist circumferences, girth measurements) and a progressive maximal effort treadmill run will be completed. The progressive treadmill run will comprise of running at a starting speed of 8km/hr, increasing in speed by 1 km/hr every 3 minutes, until the participant meets specific physiological criteria (rating of perceived exertion of 18, RER of above 1.1, maximal predicted heart rate, or a plateau is seen in their oxygen consumption), and feels they cannot complete another stage, or the test is ended by the researcher. Breath-by-breath respiratory parameters (Metamax 3B; Cortex, Germany) and heart rate (S810; Polar Finland) will be continuously recorded throughout the exercise test in order to determine peak oxygen uptake (VO_2peak_) and the running speed to be undertaken during the main experimental trials.

**Main Trials**

Participants will be familiar with carbohydrate counting and instructed to follow a recommended diet plan for 48 hours prior to testing. Participants will be required to read and sign an informed consent form and medical questionnaire which will be reviewed during each visit to the laboratory.

The participants will be required to arrive at the laboratory between 6 and 8 am on the morning of each main experimental trial. Following arrival, anthropometric variables will be collected. The participant will assume a seated and rested position whilst a 20 gauge cannula is inserted into an antecubital vein in their non-dominant arm which will be kept patent with periodic infusion of saline. After collection of resting blood samples participants will consume a standardised carbohydrate breakfast (~80g of carbohydrates, adjusted for individual body mass [1g.kg.bm^-1^]) and administer a 75% reduced rapid-acting insulin dose (for example, a participant whom normally administers 1 IU of rapid-acting insulin with 10 g of carbohydrate would administer a 2 IU of rapid-acting insulin) (Rabasa-Lhoret et al., 2001; West et al., 2010; West et al., 2011a; 2011b).

Participants will remain at rest for 60 minutes before completing 45 minutes of treadmill running (Woodway, Germany) at 70% VO_2peak_. Throughout exercise, cardio respiratory parameters such as heart rate and expired air (Metamax 3B; Cortex, Germany) will be collected to monitor exercise intensity. After completion of the exercise bout participants will be placed in a seated and rested position; after 60 minutes of recovery, participants will then consume a standardised carbohydrate based meal (~80g of carbohydrates, adjusted for body mass [1g/kg/body mass]) and administer a normal, or 50% reduced rapid-acting insulin dose (for example, a participant who normally administers1 IU of rapid-acting insulin with 10 g of carbohydrate would administer 8 and 4IU of insulin for the normal and 50% reduced insulin dose trials, respectively). After ingesting the meal, participants will then remain at rest for a further 3 hours, where blood samples will be drawn periodically every 30 minutes.

For each blood sample, 2 ml of whole blood will be collected and used for the immediate quantification of glucose and lactate (Biosen C, EKF, UK), haematocrit (Micro Haematocrit MK IV, Hawksley, UK), and haemoglobin (Haemocue limited, UK). Changes in plasma volume will be corrected for using the method of Dill and Costill (1974). Moreover, 15 ml of blood will be transferred into tubes designed to collect blood plasma and serum. Both containers will then be centrifuged at 3000 x g for 15 minutes to obtain plasma and serum, which will be stored at -80 C° for later analysis of metabolic, counter-regulatory hormonal and inflammatory parameters by enzyme immunoassay. After the initial blood samples, further samples will be taken at 30 and 60 minutes before exercise, then 0, 15, 30 and 60 minutes after exercise (see Figure 1). Following the carbohydrate meal and insulin administration, blood samples will then be collected every 30 minutes for 3 hours. The total whole blood volume collected during each trial will be 240 ml (the equivalent of approximately 48 tea spoons, 72% of a typical soft drink can; or 4% of the total volume of blood in the body). Moreover, a total whole blood volume of 720 ml (144 teaspoons; 2.2 soft drink cans) will be collected per participant over the duration of the study.

During the experimental trials breath-by-breath respiratory parameters will be collected during the 45 minute exercise period, which will facilitate the calculation of substrate oxidation rates and energy consumption using principles of indirect calorimetry. Heart rate will be continuously recorded throughout exercise.


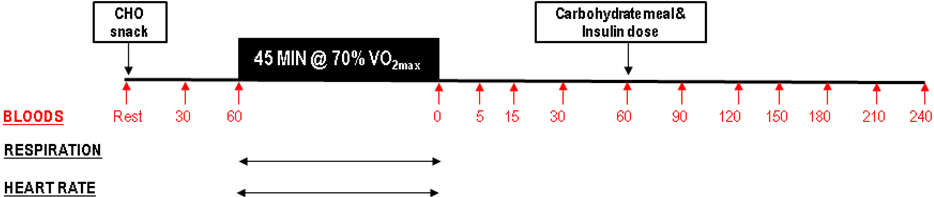


Figure 1: Within laboratory blood sampling time points.
