# Supplementary material for: Metabolic Implications when Employing Heavy Pre- and Post-Exercise Rapid-Acting Insulin Reductions to Prevent Hypoglycaemia in Type 1 Diabetes Patients: A Randomised Clinical Trial
Source: PLoS One. 2014 May 23;9(5):e97143. doi: 10.1371/journal.pone.0097143 (PMC4032262; doi:10.1371/journal.pone.0097143)
Supplement: Diagram S1 — (DOC) [file pone.0097143.s003.doc]

**CONSORT 2010 Flow Diagram**

**Allocation**

**Analysis**

**Follow-Up**

**Enrollment**

Assessed for eligibility (n= 8 )

Excluded (n= 0 )

♦  Not meeting inclusion criteria (n=0 )

♦  Declined to participate (n= 0 )

♦  Other reasons (n= 0)

N/A

N/A

N/A

Lost to follow-up (give reasons) (n= 0)

Discontinued intervention (give reasons) (n= 0 )

Allocated to intervention (n= 8 )

♦ Received allocated intervention (n=8 )

♦ Did not receive allocated intervention (give reasons) (n= 0 )

Analysed (n=8 )
♦ Excluded from analysis (give reasons) (n= 0 )

Randomized (n= 8 )
